# Phase II study of capecitabine in combination with paclitaxel in patients with anthracycline-pretreated advanced/metastatic breast cancer

**DOI:** 10.1038/sj.bjc.6601784

**Published:** 2004-04-06

**Authors:** N Batista, G Perez-Manga, M Constenla, A Ruiz, F Carabantes, J Castellanos, M Gonzalez Barón, K Villman, M Söderberg, J Ahlgren, J Casinello, P Regueiro, A Murias

**Affiliations:** 1 Hospital Universitario, Campus de Ofra, La Laguna, Tenerife E-38320, Spain; 2 Hospital Gregorio Marañón, Madrid, Spain; 3 Hospital Montecelo, Pontevedra, Spain; 4 IVO, Valencia, Spain; 5 Hospital Carlos Haya, Malaga, Spain; 6 Hospital Xeral Cies, Vigo, Spain; 7 Hospital La Paz, Madrid, Spain; 8 Orebro University Hospital, Orebro, Sweden; 9 Central Hospital of Karlstad, Sweden; 10 Academic Hospital, Uppsala, Sweden; 11 Hospital General, Guadalajara, Spain; 12 F. Hoffmann-La Roche, Madrid, Spain; 13 Hospital Insular, Las Palmas, Spain

**Keywords:** capecitabine, paclitaxel, anthracycline, breast cancer, fluoropyrimidines, oral

## Abstract

The addition of oral capecitabine to docetaxel improves response rate, time to progression (TTP) and overall survival in anthracycline-pretreated metastatic breast cancer (MBC). This phase II study evaluates the efficacy and safety of a 21-day cycle of oral capecitabine (1000 mg m^−2^ twice daily, days 1–14) plus i.v. paclitaxel (175 mg m^−2^, day 1) in anthracycline-pretreated advanced/MBC. In all, 73 patients were enrolled at 13 Swedish and Spanish centres. The objective response rate was 52% (95% confidence interval (CI): 40–63%) in the intent-to-treat population, including complete responses in 11%. Disease was stabilised in a further 29%. The median time to disease progression (TTP) was 8.1 months and the median overall survival was 16.5 months. The combination was generally well tolerated with a predictable safety profile. The most common treatment-related nonhaematological adverse events were hand–foot syndrome (42%), alopecia (30%) and diarrhoea (26%). The only treatment-related Grade 3/4 adverse events occurring in >5% of patients were alopecia (22%) and hand–foot syndrome (11%). Grade 3/4 neutropenia and lymphocytopenia were reported in 12 and 14% of patients, respectively. Capecitabine plus paclitaxel is highly active with a favourable safety profile in anthracycline-pretreated MBC.

Systemic cytotoxic chemotherapy is the treatment of choice for patients with advanced/metastatic breast cancer (MBC) that is hormone resistant, oestrogen/progesterone-receptor negative, or shows evidence of rapid progression, particularly in visceral sites. The majority of patients receive an anthracycline-based regimen as their first chemotherapy treatment for breast cancer, in either the (neo)adjuvant or metastatic setting. For patients whose disease has progressed during or following treatment with anthracyclines, single-agent docetaxel was, until recently, the standard of care, based on a survival advantage compared with mitomycin C plus vinblastine observed in a randomised phase III trial (Nabholtz *et al*, 1999). No combination regimen had been shown to improve outcomes compared with single-agent docetaxel, until a phase III study demonstrated that the addition of the oral fluoropyrimidine capecitabine (Xeloda®: F Hoffmann-La Roche, Basel, Switzerland) to docetaxel improves response rates, time to progression (TTP) and, most importantly, overall survival in patients with anthracycline-pretreated MBC (O'Shaughnessy *et al*, 2002). In light of these findings, we report here the results of a phase II study evaluating capecitabine in combination with paclitaxel (Taxol®: Bristol-Myers Squibb Company, New Jersey, USA) in patients with anthracycline-pretreated MBC.

Oral capecitabine generates 5-fluorouracil (5-FU) preferentially in tumour tissue by exploitation of the increased activity of thymidine phosphorylase (TP) in tumour compared with normal tissue (Miwa *et al*, 1998; Schüller *et al*, 2000). After oral administration, capecitabine is rapidly and virtually completely absorbed intact through the gastrointestinal wall and metabolised to 5-FU via a three-step enzymatic cascade. In the final step, 5-FU is generated preferentially at the tumour site due to the significantly higher TP activity in tumour tissue. This potentially improves efficacy and safety. Single-agent capecitabine is considered by many to be the reference treatment for patients with taxane-pretreated MBC. In four trials conducted in a total of 500 patients with anthracycline- and taxane-pretreated disease, capecitabine achieved objective response rates ranging from 15 to 28%, overall disease control rates of 57–63% and median overall survival of approximately 12 months (Blum *et al*, 1999, 2001a; Fumoleau *et al*, 2004; Reichardt *et al*, 2003). In addition, capecitabine demonstrated a favourable safety profile, with a particularly low incidence of myelosuppression, severe adverse events and alopecia.

There is a clear rationale behind the evaluation of capecitabine/taxane combinations in patients with breast cancer. With a low incidence of myelosuppression, capecitabine is an ideal agent for incorporation into combination regimens with myelosuppressive agents such as the taxanes. In addition, preclinical studies show that many cytotoxic agents, including paclitaxel and docetaxel, upregulate TP in tumour tissue and have synergistic antitumour activity when combined with capecitabine (Sawada *et al*, 1998; Ishitsuka, 2000).

Paclitaxel has demonstrated efficacy across a variety of treatment schedules and is commonly administered in patients with anthracycline-pretreated MBC (Seidman *et al*, 1995a, 1995b). A phase I study demonstrated that a 3-weekly regimen combining i.v. paclitaxel, administered on day 1 as a 3-h infusion, with oral capecitabine, administered twice daily on days 1–14, is feasible and clinically active in patients with pretreated MBC. No significant pharmacokinetic interactions between the two agents were reported (Villalona-Calero *et al*, 2001). The principal dose-limiting toxicities of the combination therapy were the cutaneous side effect hand–foot syndrome, which is characteristic of protracted infused fluoropyrimidines or capecitabine (which was designed to mimic continuous infusion of 5-FU), and neutropenia, which is dose limiting with single-agent paclitaxel.

The current phase II study was performed to evaluate the efficacy and safety of capecitabine plus 3-weekly paclitaxel in patients with anthracycline-pretreated advanced or MBC.

## PATIENTS AND METHODS

### Study design

This open-label, phase II trial of intermittent, oral capecitabine in combination with i.v. paclitaxel in patients with anthracycline-pretreated advanced/MBC was conducted between August 1998 and 2000 in accordance with the International Good Clinical Practice Principles and local ethical and regulatory requirements. All patients provided written informed consent before screening and enrolment. The primary objective of the study was to assess the antitumour activity of the capecitabine/paclitaxel regimen, determined by objective response rate. Secondary objectives were to evaluate duration of response, TTP, overall survival and safety.

Oral capecitabine 1000 mg m^−2^ was administered twice daily on days 1–14 of each 3-week treatment cycle. Paclitaxel 175 mg m^−2^ was administered as a 3-h infusion on day 1 of each treatment cycle. It was recommended that all patients receive routine prophylactic corticosteroid, antihistamine and H_2_-receptor antagonist premedication before the administration of paclitaxel. Additional permitted supportive treatment and concomitant medication were administered as required at the investigator's discretion. Haemopoietic growth factors could be used to treat symptomatic neutropenia.

Duration of treatment depended on tumour response: patients with stable disease or a partial response (PR) received a maximum of 10 cycles of chemotherapy, and patients with a complete response (CR) received four cycles of chemotherapy following confirmation of CR. Upon documentation of disease progression study treatment was discontinued.

### Patient population

The study was conducted in women with histologically confirmed, bidimensionally measurable breast cancer that had progressed during or following anthracycline-containing therapy administered in the adjuvant or metastatic setting. Patients were required to meet the following eligibility criteria: age 18–70 years, life expectancy >3 months, Karnofsky performance status (KPS) ⩾60%, absolute neutrophil count (ANC) ⩾1500 *μ*l^−1^, platelet count ⩾100 000 *μ*l^−1^, total bilirubin <1.5 × the upper normal limit (UNL), aspartate transaminase (ASAT) and/or alanine transaminase (ALAT) ⩽5 × UNL, alkaline phosphatase ⩽5 × UNL (although this was permitted if bone metastases were present and there was no evidence of a liver disorder) and serum creatinine concentration ⩽133 *μ*mol l^−1^. Patients were excluded if they had been previously exposed to paclitaxel in the adjuvant or metastatic setting and if they had previously received more than two chemotherapy regimens for treatment of metastatic disease. Prior docetaxel treatment was permitted. Previous cytotoxic chemotherapy or hormonal therapy was to have been completed >6 weeks or >10 days, respectively, prior to the initiation of study treatment. In addition, patients were to have received no transfused blood products or haematopoietic growth factors during the 2 weeks prior to the start of study treatment.

The following patients were excluded from the study: pregnant or lactating patients; women of childbearing potential who lacked a reliable method of contraception; patients with a history of seizures, CNS disorders or psychiatric disability; patients with CNS metastases; and patients who showed evidence of any other serious illness or medical condition, including serious uncontrolled infections. Patients with clinically significant cardiac disease, as defined by symptomatic ventricular arrhythmias, history of congestive heart failure or previous myocardial infarction within 12 months of study enrolment were excluded, as were patients with malabsorption syndromes or a history of previous gastrointestinal surgery affecting oral absorption. Additional exclusion criteria included a history of: organ allograft; another malignancy within 5 years of study entry (except basal cell carcinoma of the skin or carcinoma *in situ* of the uterine cervix); severe and unexpected reaction to paclitaxel (or its formulation constituents) or fluoropyrimidine therapy (with or without documented dihydropyrimidine dehydrogenase deficiency); and a known hypersensitivity to 5-FU.

At the time the study was started, data showing the impact of moderate/severe renal impairment on the safety of capecitabine were not available. However, the protocol recommended caution when administering capecitabine in patients with moderate/severe renal impairment. More recent data led to contraindication of capecitabine in patients with severe renal impairment (defined as creatinine clearance <30 ml l^−1^) and a 25% reduction of the capecitabine starting dose in patients with moderate impairment (creatinine clearance 30–50 ml l^−1^) at baseline (Cassidy *et al*, 2002).

### Dose modification

Treatment was continued at the same dose (without interruption or dose reduction) if patients experienced Grade 1 toxicities or other toxicities considered unlikely to become serious or life threatening (e.g. alopecia). For all other treatment-related adverse events with intensity of Grade 2 or higher (except Grade 3 peripheral neuropathy, severe fluid retention, hypersensitivity reactions, hepatic impairment or neutropenia, as described below), the following dose modification scheme was implemented. Dose reduction was not required following the first appearance of any Grade 2 toxicity, although treatment was interrupted/delayed until the toxicity had resolved to Grades 0–1 and symptomatic treatment was initiated when possible. Treatment with both agents was interrupted/delayed and the dose of both agents was reduced by 25% in patients who experienced a second occurrence of a given Grade 2 toxicity or at the first occurrence of a Grade 3 toxicity. If patients experienced a third occurrence of a given Grade 2 toxicity or a second occurrence of a given Grade 3 toxicity, treatment was interrupted/delayed until the toxicity resolved to Grades 0–1 and the dose of capecitabine was reduced by 50% and paclitaxel discontinued. Treatment with both agents was discontinued if, despite dose reduction, a given toxicity occurred for a fourth time at Grade 2 or a third time at Grade 3. Treatment was also discontinued if patients experienced a Grade 4 nonhaematological toxicity, unless the investigator considered it to be in the best interest of the patient to continue treatment with single-agent capecitabine at 50% of the original dose.

Paclitaxel was discontinued and capecitabine treatment was modified according to the scheme outlined above in patients experiencing Grade 3 peripheral neuropathy or severe fluid retention (e.g. pleural effusion, pericardial effusion or ascites). Patients developing severe hypersensitivity reactions (e.g. blood pressure decreased by ⩾20 mg m^−2^, bronchospasm or generalised rash/erythema) during or following paclitaxel administration were taken off study immediately and given appropriate therapy. In patients developing Grade 2 elevations in transaminase (ASAT and/or ALAT) concentrations with a Grade 2 elevation in alkaline phosphatase concentration, the dose of paclitaxel was reduced by 25%. Paclitaxel treatment was interrupted in patients experiencing Grade 3 elevations in these enzymes and discontinued if the liver function had not recovered within 2 weeks of the interruption.

In the case of neutropenia, paclitaxel was readministered only when ANC recovered to ⩾1.5 × 10^9^ l^−1^. In patients experiencing Grade 4 neutropenia (ANC ⩽0.5 × 10^9^ l^−1^) for more than 7 days or febrile (⩾38°C) neutropenia, the dose of paclitaxel was reduced by 25%. Paclitaxel was discontinued if patients receiving the reduced dose experienced Grade 4 neutropenia or febrile neutropenia. As capecitabine was not expected to worsen or prolong neutropenic episodes, capecitabine treatment could be continued during episodes of Grade 3–4 neutropenia. However, capecitabine was to be interrupted upon the development of any other Grade 2 toxicity during the neutropenic episode, with the patient closely monitored in hospital. The next cycle was not started until neutropenia had resolved to Grades 0–1.

### Patient evaluation

Tumour evaluation, based on WHO criteria (WHO, 1979), was performed at baseline and at 6-weekly intervals during the study period. The best overall response was defined as the best response recorded from the start of treatment to disease progression. Complete response and PR were confirmed by a second tumour assessment after ⩾4 weeks. For patients demonstrating an objective response, the time to response was defined as the interval between the start of treatment and the first observation of response. In patients achieving a CR or PR, the duration of response was defined as the time interval between the start of treatment and the first observation of disease progression or, in patients with no documented disease progression, death or last contact. Time to progression was defined as the interval between the start of treatment and disease progression (or death or last contact in patients with no documented disease progression) in all patients. Overall survival was defined as the interval between the start of treatment and death.

Safety was evaluated in all patients receiving at least one dose of study treatment. Adverse events (with the exception of hand–foot syndrome) and abnormal laboratory parameters were graded according to the National Cancer Institute of Canada Common Toxicity Grade (NCIC CTG). Hand–foot syndrome was graded 1–3 using established criteria (Blum *et al*, 1999; Blum, 2001). The nature, severity and outcome of all adverse events occurring during treatment were recorded.

### Statistical analysis

Sample size was calculated based on the method of Fleming (1982). In advanced breast cancer, a response rate of 20% can be considered of little interest, while 40% or more can be considered encouraging. With a power of 95% and a one-sided significance level of 5%, a total of 54 patients was required. Assuming a 15% dropout rate, a target of 64 patients for recruitment was adopted.

All efficacy analyses were performed using the ITT population. Time to progression, duration of response and overall survival were estimated by Kaplan–Meier analysis.

## RESULTS

### Patient demographics and baseline disease characteristics

Patient characteristics are summarised in Table 1

**Table 1:** Patient and baseline disease characteristics (*n*=73)

Median age (years) (range)	52 (33–74)
*KPS, no.* (%) *of patients*	
100	31 (42)
90	19 (26)
80	15 (21)
70	7 (10)
60	1 (1)
	
*Number of metastatic sites, no.* (%) *of patients*^a^	
1	26 (36)
2	25 (34)
⩾3	21 (29)
	
*Sites of metastasis, no.* (%) *of patients*^a^	
Bone	33 (45)
Liver	32 (44)
Lymph nodes	29 (40)
Lung	25 (34)
	
*Prior chemotherapy*	
Anthracyclines, no. (%) of patients	73 (100)
(Neo)adjuvant setting	42 (58)
Metastatic setting	34 (47)
Docetaxel, no. (%) of patients	11 (15)
Cyclophosphamide, no. (%) of patients	38 (52)
5-FU, no. (%) of patients	31 (42)

a Confirmation of metastatic disease was not available in one patient. KPS=Karnofsky performance status; 5-FU=5-fluorouracil.

. A total of 73 patients with a median age of 52 years (range 33–74 years) were enrolled in the study at 13 centres in Spain and Sweden. The median KPS was 90% (range 60–100%), with KPS ⩾80% in the majority of patients (89%). Approximately two-thirds of the patients (63%) had multiple sites of metastasis. The most common metastatic sites were bone (45% of patients), liver (44%), lymph nodes (40%) and lung (34%).

As predefined in the protocol, all patients had received prior anthracycline therapy. Of the 34 patients pretreated with anthracyclines in the metastatic setting, three had also received neoadjuvant or adjuvant anthracyclines.

A total of 512 cycles of chemotherapy (combination or capecitabine monotherapy) were administered, with a median of eight treatment cycles per patient (range 1–11). All 73 patients received at least one dose of study therapy and were therefore evaluable for safety. The majority of patients who withdrew from treatment with either agent stopped receiving both treatments, so that very few patients received either capecitabine or paclitaxel monotherapy during any cycle (Table 2

**Table 2:** Patients receiving capecitabine plus paclitaxel combination therapy or monotherapy with either agent at each cycle

**Cycle number**	**Combination therapy**	**Capecitabine monotherapy**	**Paclitaxel monotherapy**	**Off study**
1	72	0	1	0
2	69	0	0	4
3	63	0	1	9
4	59	0	0	14
5	53	0	2	18
6	48	1	2	22
7	41	0	3	29
8	34	1	3	35
10	27	1	1	44

No data available for cycle 9.

). One patient received paclitaxel monotherapy and withdrew during the first cycle without receiving capecitabine.

### Efficacy

Overall, the capecitabine/paclitaxel regimen demonstrated an objective response rate of 52% (95% CI: 40–63), with CR in eight patients (11%) and PR in 30 patients (41%) (Table 3

**Table 3:** Antitumour activity (Intent-to-treat population, *n*=73)

	**No. of patients**	**% of patients (95% CI)**
Objective response	38	52 (40–63)
CR	8	11 (5–21)
PR	30	41 (29–53)
Stable disease	21	29 (20–43)

CI=confidence interval; CR=complete response; partial response.

). Disease was stabilised in a further 21 patients (29%). Retrospective analyses revealed no differences in response rates between subpopulations: response rates were similarly high in patients pretreated with anthracyclines in the (neo)adjuvant setting only (*n*=39) (59%; 95% CI: 42–74) and metastatic setting (*n*=34) (42%; 95% CI: 26–61). Of the 11 patients pretreated with docetaxel in the metastatic setting, four (36%) achieved an objective response and disease was stabilised in a further five patients (45%).

**Figure 1:**
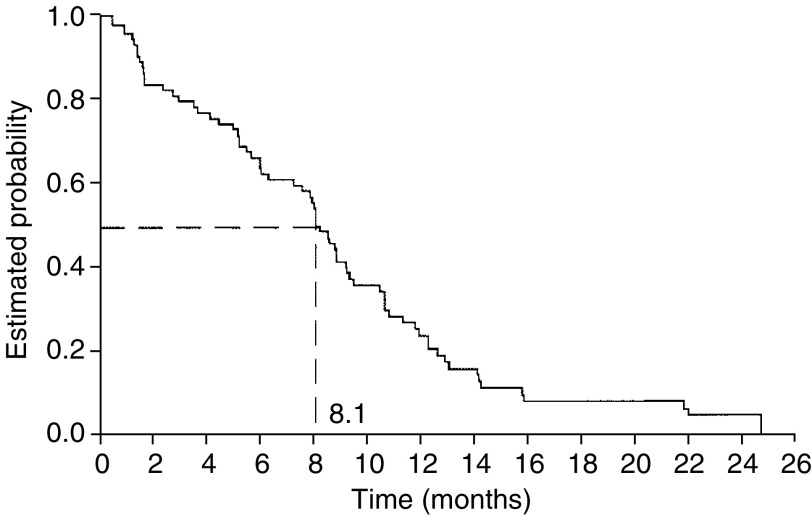
Time to disease progression or death.

**Figure 2:**
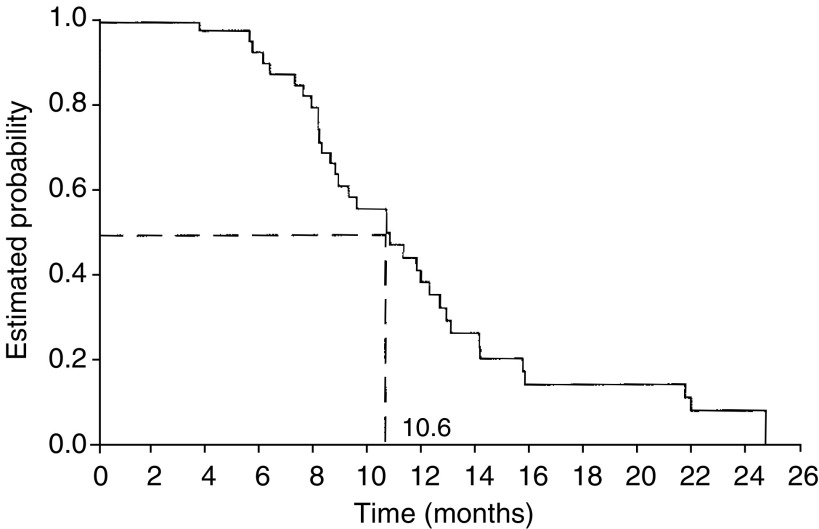
Duration of response.

**Figure 3:**
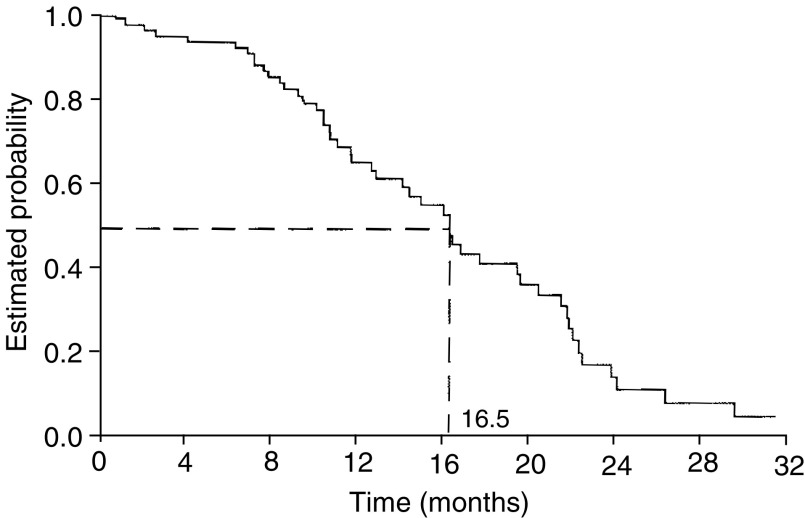
Overall survival.

The median follow-up at the time of data analysis was 8.0 months (range 0.2–24.5 months) and disease progression or death had occurred in 54 patients during this period. The median TTP for all patients was 8.1 months (95% CI: 6.2–9.2) (Figure 1

) and median duration of response in the 38 responding patients was 10.6 months (95% CI: 8.7–11.4) (Figure 2

). Median overall survival was 16.5 months (95% CI: 12.6–20.0) (Figure 3

).

### Safety

Apart from alopecia, which is commonly associated with paclitaxel-containing therapies and occurred in a substantial proportion of patients (30%), the most frequent (>20% of patients) treatment-related, clinical adverse events were hand–foot syndrome (42%), diarrhoea (26%), myalgia (25%), vomiting (23%), neurotoxicity (21%) and asthenia (21%) (Table 4

**Table 4:** Most frequent (affecting >10% patients) treatment-related clinical adverse events (*n*=73)

	**NCIC-CTG grade: no. (%) of patients**		
	**Grade 1/2**	**Grade 3**	**Grade 4**
Hand–foot syndrome^a^	23 (32)	8 (11)	—
Alopecia	6 (8)	16 (22)	—
Diarrhoea	17 (23)	1 (1)	1 (1)
Myalgia	18 (25)	—	—
Vomiting	15 (21)	1 (1)	1 (1)
Neurotoxicity	13 (18)	2 (3)	—
Asthenia	15 (21)	—	—
Nausea	11 (15)	—	—
Paraesthesia	9 (12)	1 (1)	—
Mucositis	6 (8)	1 (1)	1 (1)

NCIC CTG=National Cancer Institute of Canada Common Toxicity Grade.

a Graded 1–3 using established criteria (Blum 2001; Blum *et al*, 1999).

). The majority of treatment-related adverse events were mild to moderate in intensity. The only treatment-related Grade 3/4 clinical adverse events occurring in more than 5% of patients were alopecia (22%, Grade 3 only) and hand–foot syndrome (11%, Grade 3 only). Grade 3/4 treatment-related diarrhoea occurred in only two patients (Grade 3 in one patient, Grade 4 in the other). Grade 3/4 neurotoxicity was also rare with the capecitabine/paclitaxel combination: only one patient experienced Grade 4 peripheral neuropathy and there was only one case of Grade 3 paraesthesia.

The most common Grade 4 haematological abnormality was neutropenia, which occurred in 5% of patients (Table 5

**Table 5:** Incidence of treatment-related Grade 3/4 haematological adverse events (*n*=73)

	**NCIC-CTC grade: no. (%) of patients**	
**Adverse event**	**Grade 3**	**Grade 4**
Lymphocytopenia	7 (10)	3 (4)
Neutropenia	5 (7)	4 (5)
Leucopenia	7 (10)	2 (3)
Neutropenic fever	2 (3)	1 (1)
Thrombocytopenia	1 (1)	1 (1)

NCIC CTC=National Cancer Institute of Canada Common Toxicity Criteria.

). Grade 3/4 lymphocytopenia occurred in 10 patients (14%). There was a low incidence of Grade 3/4 thrombocytopenia (two patients, 3%) and Grade 3/4 anaemia was not observed. Febrile neutropenia was reported in only three patients (4%). Two patients received G-CSF for the treatment of neutropenia (one patient with Grade 3 leucopenia and Grade 2 fever and the other with Grade 3 febrile neutropenia). In addition, one patient received prophylactic G-CSF.

A total of 44 patients (60%) withdrew from the study before completion of treatment, more than half (23 patients, 52%) due to disease progression. A further 13 patients (30%) withdrew due to adverse events. No treatment-related deaths occurred during the study, but two patients died due to disease progression before completing treatment.

## DISCUSSION

Currently, the taxanes are the most widely used agents for the treatment of patients with anthracycline-pretreated advanced/MBC. Paclitaxel was the first taxane to show activity in MBC (Holmes *et al*, 1991) and has demonstrated activity in anthracycline-pretreated patients in a number of phase II trials, with response rates varying from 17 to 53% (Abrams *et al*, 1995; Dieras *et al*, 1995; Gianni *et al*, 1995; Fountzilas *et al*, 1996; Nabholtz *et al*, 1996; Holmes *et al*, 1998; Seidman *et al*, 1998; Smith *et al*, 1999). The efficacy of single-agent paclitaxel has not been definitively evaluated *vs* a standard salvage therapy in a randomised, phase III trial, however. Although paclitaxel has demonstrated efficacy in phase III trials in the first-line setting (Paridaens *et al*, 2000; Sledge *et al*, 2003), the optimal dose schedule for single-agent administration has not been fully established. The most widely used regimen is a 3-weekly schedule of 135–175 mg m^−2^, although weekly administration of paclitaxel has also been evaluated (Seidman *et al*, 1998). With the weekly schedule, there was relatively little myelosuppression, but peripheral neuropathy prevented dose escalation above 100 mg m^−2^.

Attempts to increase the efficacy of paclitaxel have largely focused on its use in combination regimens. However, the side effect profile of paclitaxel, which includes a high rate of myelosuppression, has limited its use in combination regimens with other myelosuppressive drugs. Since capecitabine is associated with a very low incidence of myelosuppression, its use as a combination partner for paclitaxel in the current study (along with the administration of G-CSF support) may explain the relatively low incidence of severe myelosuppression experienced by patients.

The current study shows that capecitabine in combination with 3-weekly paclitaxel is highly active in patients with anthracycline-pretreated MBC, consistent with the findings of O'Shaughnessy *et al* (2002) with capecitabine plus docetaxel. The present regimen resulted in a high rate of disease control in these patients, with objective responses in 52% of patients and disease stabilisation in a further 29%. Furthermore, objective response rates appear similar in the subpopulations of patients pretreated with anthracyclines only in the (neo)adjuvant setting (59%) or in the metastatic setting (42%). Although there was a trend toward a higher response rate in the subpopulation pretreated with (neo)adjuvant anthracyclines, the difference was not statistically significant. In addition to the impressive rate of tumour control, time-dependent outcomes with the combination were very encouraging, with a median TTP of 8.1 months, a median duration of response of 10.6 months and a median overall survival of 16.5 months.

Capecitabine/paclitaxel combination therapy is generally well tolerated, demonstrating a predictable safety profile consistent with the safety profiles of the component agents. The most common treatment-related adverse event was hand–foot syndrome, a cutaneous side effect that is characteristic of prolonged cytostatic administration. The majority of cases of hand–foot syndrome were mild to moderate in intensity, and only eight patients (11%) experienced severe hand–foot syndrome. The incidence of hand–foot syndrome was thus in a similar range to that observed by Blum *et al* (1999) with capecitabine monotherapy (1250 mg m^−2^ twice daily). Previous studies have shown that this side effect is readily manageable with capecitabine treatment interruption and dose modification, if necessary (Blum, 2001; Blum *et al*, 2001b). Based on the safety profiles of single-agent capecitabine and paclitaxel, gastrointestinal side effects were also anticipated with the combination. However, fewer episodes of diarrhoea, nausea and vomiting occurred than are usually seen with standard-dose monotherapy with either agent. These were not problematic and were effectively managed by dose modification, supportive care and pharmacological interventions.

Grade 3/4 treatment-related diarrhoea and vomiting were each reported by only two patients. Neurological side effects have been frequently reported with paclitaxel, although in our study, asthenia generally occurred at only mild–moderate intensity. Severe (Grade 3) neurotoxicity was reported in only two patients (3%). Neutropenia, which is also commonly associated with paclitaxel treatment, was effectively managed in the majority of cases (including the curative use of G-CSF in two patients). Continuation of capecitabine therapy in patients with Grade 3/4 neutropenia did not appear to compromise haematological recovery.

The results of the current study are supported by the preliminary data of a phase II study evaluating a similar regimen (capecitabine 825 mg m^−2^ twice daily, days 1–14 in combination with paclitaxel 175 mg m^−2^ on day 1) in patients with predominantly untreated MBC (Gradishar *et al*, 2003). In this study, an objective response rate of 51% was reported, almost identical to that observed in the current study, with a median TTP of 10.6 months and a median overall survival of 29.9 months. The long overall survival possibly reflects the fact that patients had received less extensive pretreatment than those in the current study. A similar safety profile was observed in both studies. A phase I/II trial is also evaluating the feasibility of capecitabine with weekly paclitaxel.

Paclitaxel has also been studied in combination with gemcitabine *vs* paclitaxel alone as first-line treatment for MBC in a phase III study (O'Shaughnessy *et al*, 2003). All patients were pretreated with anthracyclines in the adjuvant setting. Gemcitabine plus paclitaxel showed disappointing results achieving an overall response rate of 39% compared with 26% with paclitaxel alone. The median TTP was 5.4 months with gemcitabine/paclitaxel *vs* 3.5 months with paclitaxel alone. The median overall survival has not been reached. The gemcitabine/paclitaxel combination does not appear to be as effective as the capecitabine/paclitaxel combination (response rate of 52%, median TTP 8.1 months) presented here.

The efficacy of the capecitabine/paclitaxel combination is very promising, comparing favourably with that reported for capecitabine/docetaxel in anthracycline-pretreated MBC (response rate 42%, median TTP 6.1 months and median overall survival 14.5 months) (O'Shaughnessy *et al*, 2002). Further evaluation of the capecitabine/paclitaxel combination *vs* capecitabine/docetaxel, which is now considered the ‘gold standard’ chemotherapy for anthracycline-pretreated MBC, is warranted.

In summary, the results of this phase II study demonstrate that capecitabine plus paclitaxel is highly effective with a favourable safety profile in patients with anthracycline-pretreated advanced/MBC. Phase III evaluation of this regimen is now required to establish definitively the efficacy of this combination in patients with advanced/MBC.

## References

[bib1] Abrams JS, Vena DA, Baltz J, Adams J, Montello M, Christian M, Onetto N, Desmond-Hellmann S, Canetta R, Friedman MA, Arbuck SG (1995) Paclitaxel activity in heavily pretreated breast cancer: a National Cancer Institute Treatment Referral Center trial. J Clin Oncol 13: 2056–2065 7543562 10.1200/JCO.1995.13.8.2056

[bib2] Blum JL (2001) The role of capecitabine, an oral, enzymatically activated fluoropyrimidine, in the treatment of metastatic breast cancer. Oncologist 6: 56–64 11161228 10.1634/theoncologist.6-1-56

[bib3] Blum JL, Dieras V, Lo Russo PM, Horton J, Rutman O, Buzdar A, Osterwalder B (2001a) Multicenter, phase II study of capecitabine in taxane-pretreated metastatic breast carcinoma patients. Cancer 92: 1759–1768 11745247 10.1002/1097-0142(20011001)92:7<1759::aid-cncr1691>3.0.co;2-a

[bib4] Blum JL, Jones SE, Buzdar AU (2001b) Capecitabine in 162 patients with paclitaxel-pretreated MBC: updated results and analysis of dose-modification. Eur J Cancer 37(Suppl 6): 190 (abstr. 693)

[bib5] Blum JL, Jones SE, Buzdar AU, Lo Russo PM, Kuter I, Vogel C, Osterwalder B, Burger HU, Brown CS, Griffin T (1999) Multicenter phase II study of capecitabine in paclitaxel-refractory metastatic breast cancer. J Clin Oncol 17: 485–493 10080589 10.1200/JCO.1999.17.2.485

[bib6] Cassidy J, Twelves C, Van Cutsem E, Hoff P, Bajetta E, Boyer M, Bugat R, Burger U, Garin A, Graeven U, McKendrick J, Maroun J, Marshall J, Osterwalder B, Pérez-Managa G, Rosso R, Rougier P, Schilsky RL (2002) First-line oral capecitabine therapy in metastatic colorectal cancer: a favorable safety profile compared with intravenous 5-fluorouracil/leucovorin. Ann Oncol 13: 566–575 12056707 10.1093/annonc/mdf089

[bib7] Dieras V, Marty M, Tubiana N, Corette L, Morvan F, Serin D, Mignot L, Chazard M, Garet F, Onetto N, Hellmann S, Pouillart P (1995) Phase II randomized study of paclitaxel versus mitomycin in advanced breast cancer. Semin Oncol 22: 33–39 7638640

[bib8] Fleming TR (1982) One-sample multiple testing procedure for phase II clinical trials. Biometrics 38: 143–151 7082756

[bib9] Fountzilas G, Athanassiades A, Giannakakis T, Bafaloukos D, Karakousis K, Dombros N, Kosmidis P, Skarlos D (1996) A phase II study of paclitaxel in advanced breast cancer resistant to anthracyclines. Eur J Cancer 32: 47–51 10.1016/0959-8049(95)00398-3 8695240

[bib10] Fumoleau P, Largillier R, Clippe C, Dièras V, Orfeuvre H, Lesimple T, Culine S, Audhuy B, Serin D, Curé H, Vuillemin E, Morère J-F, Montestruc F, Mouri Z, Namer M (2004) Multicentre, phase II study evaluating capecitabine monotherapy in patients with anthracycline- and taxane-pretreated metastatic breast cancer. Eur J Cancer 40: 536–542 14962720 10.1016/j.ejca.2003.11.007

[bib11] Gianni L, Munzone E, Capri G, Villani F, Spreafico C, Tarenzi E, Fulfaro F, Caraceni A, Martini C, Laffranchi A (1995) Paclitaxel in metastatic breast cancer: a trial of two doses by a 3-hour infusion in patients with disease recurrence after prior therapy with anthracyclines. J Natl Cancer Inst 87: 1169–1175 7674322 10.1093/jnci/87.15.1169

[bib12] Gradishar W, Meza L, Hill T, Samid D, Chen Y-M, Amin B (2003) A multicenter phase II study of capecitabine plus paclitaxel in metastatic breast cancer: survival update. Eur J Cancer 1(Suppl. 5): S141 (abstr. 463)

[bib13] Holmes FA, Valero V, Buzdar AU, Booser DJ, Winn R, Tolcher A, Seidman A, Goodwin W, Bearden J, Baysinger L, Hortobagyi GN, Arbuck SA (1998) Final results: randomized phase II trial of paclitaxel by 3-hr versus 96-hr infusion in patients (pt) with met breast cancer (MBC). The long & short of it. Proc Am Soc Clin Oncol 17: 110a (abstr. 426)

[bib14] Holmes FA, Walters RS, Theriault RL, Forman AD, Newton LK, Raber MN, Buzdar AU, Frye DK, Hortobagyi GN (1991) Phase II trial of taxol, an active drug in the treatment of metastatic breast cancer. J Natl Cancer Inst 83: 1797–1805 1683908 10.1093/jnci/83.24.1797-a

[bib15] Ishitsuka H (2000) Capecitabine: preclinical pharmacology studies. Invest New Drugs 18: 343–354 11081570 10.1023/a:1006497231579

[bib16] Miwa M, Ura M, Nishida M, Sawada N, Ishikawa T, Mori K, Shimma N, Umeda I, Ishitsuka H (1998) Design of a novel oral fluoropyrimidine carbamate, capecitabine, which generates 5-fluorouracil selectively in tumours by enzymes concentrated in human liver and cancer tissue. Eur J Cancer 34: 1274–1281 9849491 10.1016/s0959-8049(98)00058-6

[bib17] Nabholtz JM, Gelmon K, Bontenbal M, Spielmann M, Catimel G, Conte P, Klaassen U, Namer M, Bonneterre J, Fumoleau P, Winograd B (1996) Multicenter, randomized comparative study of two doses of paclitaxel in patients with metastatic breast cancer. J Clin Oncol 14: 1858–1867 8656254 10.1200/JCO.1996.14.6.1858

[bib18] Nabholtz JM, Senn HJ, Bezwoda WR, Melnychuk D, Deschenes L, Douma J, Vandenberg TA, Rapoport B, Rosso R, Trillet-Lenoir V, Drbal J, Molino A, Nortier JW, Richel DJ, Nagykalnai T, Siedlecki P, Wilking N, Genot JY, Hupperets PS, Pannuti F, Skarlos D, Tomiak EM, Murawsky M, Alakl M, Aapro M (1999) Prospective randomized trial of docetaxel versus mitomycin plus vinblastine in patients with metastatic breast cancer progressing despite previous anthracycline-containing chemotherapy. 304 Study Group. J Clin Oncol 17: 1413–1424 10334526 10.1200/JCO.1999.17.5.1413

[bib19] O'Shaughnessy JA, Nag S, Calderillo-Ruiz G, Jordaan J, Llombart A, Pluzanska A, Pawlicki M, Reyes JM, Sekhon J, Albain KS (2003) Gemcitabine plus paclitaxel (GT) versus paclitaxel (T) as first-line treatment for anthracycline pre-treated metastatic breast cancer (MBC): interim results of a global phase III study. Proc Am Soc Clin Oncol 22: 7 (abstr. 25)

[bib20] O'Shaughnessy J, Miles D, Vukelja S, Moiseyenko V, Ayoub JP, Cervantes G, Fumoleau P, Jones S, Lui WY, Mauriac L, Twelves C, Van Hazel G, Verma S, Leonard R (2002) Superior survival with capecitabine plus docetaxel combination therapy in anthracycline-pretreated patients with advanced breast cancer: phase III trial results. J Clin Oncol 20: 2812–2823 12065558 10.1200/JCO.2002.09.002

[bib21] Paridaens R, Biganzoli L, Bruning P, Klijn JG, Gamucci T, Houston S, Coleman R, Schachter J, Van Vreckem A, Sylvester R, Awada A, Wildiers J, Piccart M (2000) Paclitaxel versus doxorubicin as first-line single-agent chemotherapy for metastatic breast cancer: a European Organization for Research and Treatment of Cancer randomized study with crossover. J Clin Oncol 18: 724–733 10673513 10.1200/JCO.2000.18.4.724

[bib22] Reichardt P, von Minckwitz G, Thuss-Patience PC, Jonat W, Kölbl H, Jänicke F, Kieback DG, Kuhn W, Schindler AE, Mohrmann S, Kaufmann M, Lück HJ (2003) Multicenter phase II study of oral capecitabine (Xeloda®) in patients with metastatic breast cancer, relapsing after treatment with a taxane-containing therapy. Ann Oncol 14: 1227–1233 12881384 10.1093/annonc/mdg346

[bib23] Sawada N, Ishikawa T, Fukase Y, Nishida M, Yoshikubo T, Ishitsuka H (1998) Induction of thymidine phosphorylase activity and enhancement of capecitabine efficacy by Taxol/Taxotere in human cancer xenografts. Clin Cancer Res 4: 1013–1019 9563897

[bib24] Schüller J, Cassidy J, Dumont E, Roos B, Durston S, Banken L, Utoh M, Mori K, Weidekamm E, Reigner B (2000) Preferential activation of capecitabine in tumor following oral administration to colorectal cancer patients. Cancer Chemother Pharmacol 45: 291–297 10755317 10.1007/s002800050043

[bib25] Seidman AD, Hudis CA, Albanel J, Tong W, Tepler I, Currie V, Moynahan ME, Theodoulou M, Gollub M, Baselga J, Norton L (1998) Dose-dense therapy with weekly 1-hour paclitaxel infusions in the treatment of metastatic breast cancer. J Clin Oncol 16: 3353–3361 9779712 10.1200/JCO.1998.16.10.3353

[bib26] Seidman AD, Reichman BS, Crown JP, Yao TJ, Currie V, Hakes TB, Hudis CA, Gilewski TA, Baselga J, Forsythe P (1995a) Paclitaxel as second and subsequent therapy for metastatic breast cancer: activity independent of prior anthracycline response. J Clin Oncol 13: 1152–1159 7537798 10.1200/JCO.1995.13.5.1152

[bib27] Seidman AD, Tiersten A, Hudis C, Gollub M, Barrett S, Yao TJ, Lepore J, Gilewski T, Currie V, Crown J (1995b) Phase II trial of paclitaxel by 3-hour infusion as initial and salvage therapy for metastatic breast cancer. J Clin Oncol 13: 2575–2581 7595709 10.1200/JCO.1995.13.10.2575

[bib28] Sledge GW, Neuberg D, Bernardo P, Ingle JN, Martino S, Rowinsky EK, Wood WC (2003) Phase III trial of doxorubicin, paclitaxel and the combination of doxorubicin and paclitaxel as front-line chemotherapy for metastatic breast cancer: an intergroup trial (E1193). J Clin Oncol 21: 588–592 12586793 10.1200/JCO.2003.08.013

[bib29] Smith RE, Brown AM, Mamounas EP, Anderson SJ, Lembersky BC, Atkins JH, Shibata HR, Baez L, DeFusco PA, Davila E, Tipping SJ, Bearden JD, Thirlwell MP (1999) Randomized trial of 3-hour versus 24-hour infusion of high dose paclitaxel in patients with metastatic or locally advanced breast cancer: National Surgical Adjuvant Breast and Bowel Project Protocol B-26. J Clin Oncol 17: 3403–3411 10550134 10.1200/JCO.1999.17.11.3403

[bib30] Villalona-Calero MA, Blum JL, Jones SE, Diab S, Elledge R, Khoury P, Von Hoff D, Kraynak M, Moczygemba J, Kromelis P, Griffin T, Rowinsky EK (2001) A phase I and pharmacokinetic study of capecitabine and paclitaxel in breast cancer patients. Ann Oncol 12: 605–614 11432617 10.1023/a:1011181010669

[bib31] WHO Handbook for Reporting Results of Cancer Treatment (1979) WHO Offset Publication No. 48. World Health Organization, Geneva

